# Clinical excellence in nephrology: Examples from the published literature

**DOI:** 10.1186/s12882-015-0134-1

**Published:** 2015-08-15

**Authors:** Duvuru Geetha, Steven K. Lee, Amar J. Srivastava, Edward S. Kraus, Scott M. Wright

**Affiliations:** Department of Medicine, Johns Hopkins Bayview Medical Center, Johns Hopkins University, 301 Mason Lord Drive, Baltimore, MD 21224 USA

**Keywords:** Clinical excellence, Renal medicine, Nephrology

## Abstract

**Background:**

Provision of exceptional medical care is a goal for the medical profession because this is what the public needs and deserves. Academic medical centers that value excellent clinicians may have the best chance to recruit and retain these faculty members. When our institution hoped to launch the Miller Coulson Academy of Clinical Excellence to measure and reward master clinicians, a critical first step was to use rigorous methods to develop a definition of clinical excellence. Published papers have illustrated that this general definition of clinical excellence is applicable to fields of psychiatry, cardiology, and pediatrics.

**Summary:**

In this manuscript, we apply the definition of clinical excellence to nephrology. Using the same framework, we reviewed the literature to find clinical cases and exemplary nephrologists that highlight the specific domains. This collection of reports in nephrology illustrates that the definition of clinical excellence set forth by the Miller Coulson Academy is highly applicable to physicians caring for individuals with kidney disease. Relating the definition of clinical excellence to renal medicine is worthwhile in that it can help to exemplify the model to which physicians and trainees may seek to aspire.

**Key message:**

Many examples of clinical excellence in renal medicine can be found in the published medical literature. The domains of clinical excellence, described by the Miller-Coulson Academy of Clinical Excellence, apply very well to the field of nephrology.

## Introduction

Research, education, and patient care encompass the tripartite missions of academic medical centers. While the accomplishments of researchers are easily measured in terms of papers published and grants secured, those engaged primarily in patient care find it more difficult to demonstrate their success because of the lack of valid metrics that are universally accepted. The Accreditation Council for Graduate Medical Education (ACGME) has described six “core competencies” in which resident trainees must demonstrate proficiency: patient care, medical knowledge, interpersonal and communication skills, professionalism, systems-based practice, and practice-based learning and improvement [1]. However, there was little guidance in the literature that was applicable to those who had finished their training or that might help to discriminate between good and great clinicians. Defining clinical excellence is the first step to measuring clinical performance. The Miller Coulson Academy of Clinical Excellence at Hopkins used multiple methods over 2 years to identify seven domains of clinical excellence [2]. While these domains are thought to apply broadly to the practice of medicine, this article seeks to consider clinical excellence in nephrology using this framework.

The field of nephrology has experienced unprecedented growth during the past two decades due to the rising number of patients diagnosed with end stage renal disease. A substantial percentage of patients with chronic kidney disease have co-morbidities that require collaboration with different specialists and primary care physician. Nephrology currently faces several critical challenges including work force shortages, declining interest in nephrology as a career choice among trainees in internal medicine, and multiple requirements that are mandated for meaningful use credit. Many of these regulations demand a shift in management attention from the individual patient to populations, and transfer responsibilities of best practice from the single physician to groups of providers and larger entities, such as Accountable Care Organizations. As health care moves into an era of electronic medical record with demands for charting and greater documentation requirements, accountable care and bundling it is even more important to align physicians with the goals and priorities of the hospital and health care system. In addition, the financial pressure to meet RVU requirements, pressures to improve health care quality and safety and the increasingly complex logistics of the practicing environment have upped the ante for a clinician’s involvement.

While descriptions of optimal clinical performance and honorific standards have been described in other disciplines of medicine, clinical excellence in renal medicine has not been well documented in the literature. Defining clinical excellence in any medical field can serve to advance standards by holding up the ideals for how to optimally care for patients. This manuscript presents published examples, most of which are case reports that illustrate the behaviors and skills of nephrologists as they relate to the definition of clinical excellence. While some might argue that case reports are considered to be low level of medical evidence, the rationale for using them is to highlight exemplary role models in nephrology who have demonstrated clinical excellence, often in the face of challenging clinical contexts. Although witnessing excellence in person may be most inspiring, reading about brilliance and reflecting on what can be learned from inspirational role models may also be an effective means for upgrading skills.

## Definition of clinical excellence

Multiple methodological approaches were used to define clinical excellence by those responsible for developing the Miller-Coulson Academy of Clinical Excellence (MCACE) [2]. The following definition for clinical excellence, a product of an iterative process spanning more than 1 year, is used for all MCACE related initiatives.

- Achieving a level of mastery in 6 areas as they relate to patient care:
  - communication and interpersonal skills
  - professionalism and humanism
  - diagnostic acumen
  - skillful negotiation of the healthcare system
  - knowledge
  - scholarly approach to clinical practice, and
  - Exhibiting a passion for clinical medicine
- Explicitly modeling this mastery to medical trainees
- Collaborating with investigators to advance science and discovery.

The last two components of the definition relate to education and investigation so that they are primarily of relevance to clinicians in academic medical settings. The other domains are applicable to direct patient care and are relevant to all clinicians.

## Survey of the literature and selection of examples to be highlighted

A search of the literature was conducted with the assistance of a medical informationist. The search encompassed PubMed and Scopus with the parameters including “1960 through July, 2014”, “English”, and “human”. Initially, a broad search was applied using the terms ‘clinical excellence’, ‘renal medicine’, and ‘nephrology’. The search parameters were then narrowed and limited to case reports. Further searches, replaced the term ‘clinical excellence’ with each of the domains of clinical excellence listed above (e.g. communication and interpersonal skills). Using these parameters and keywords, 102 published case reports and studies were identified. The titles, abstracts, and summaries were used to sift through the published papers. Articles that appeared relevant to excellence in nephrology were evaluated in order to fully understand the care that was delivered. In addition, the references of each article were examined, adding another 127 abstracts for review. The review of these sources yielded many articles (at least ten) related to each domain of clinical excellence.

## Discussion

### Application of the definition of clinical excellence to nephrology

To illustrate how mastery in each of these domains is exemplified in renal medicine, the authors arrived at decisions by consensus about which reports from the literature would be highlighted. Each case or study demonstrates how mastery of a particular domain of clinical excellence enables clinicians to provide the best possible care for patients.

### Communication and interpersonal skills

Communication and interpersonal skills have long been hailed as cornerstones of the physician-patient relationship and clinical excellence. Nephrologists who display excellence in communication and interpersonal skills are able to clearly and effectively communicate complex plans of care with people of all different levels, acknowledge, act upon and resolve very difficult patient and family questions. The significance of these domains within the field of nephrology is highlighted in a study that examines the relationship between patient psychosocial factors and behavioral compliance in hemodialysis (HD) patients [3]. In this study, 79 HD patients were evaluated for compliance with HD treatment as well as for perception of satisfaction with all aspects of their care using the validated Patient Satisfaction Questionnaire [4], and an embedded ‘Communication and Affect’ scale [3]. Analysis of this data discovered significant associations between behavioral compliance and satisfaction with the nephrologist. It was believed that good relationships and effective communication between the nephrologist and the patient, above all else, were vital to promoting compliance and improved patient outcomes.

This conclusion was especially apparent in the case of a 48-year old Australian Aboriginal woman who was admitted to a local hospital with end stage renal disease. She ultimately declined hemodialysis treatment because a relative had died of chronic kidney disease (CKD), and she left against medical advice [5]. The renal outreach team agreed that neither their concerns nor the treatment plans had been appropriately communicated to the patient. Therefore, the team made the decision to drive 6 h to the patient’s home and discuss the situation again with the patient and her family. The patient again refused dialysis stating she wanted to remain at home close to her family but this time she did so with a full understanding of the ramifications. The community primary care team was provided with necessary education and tools to manage her ESRD. She was ultimately referred to the palliative care service. The enhanced relationship permitted the team to remain involved in her care remotely and help to maintain the patient’s quality of life well into her final days. Considered with the study above, it is evident why communication and interpersonal skills are such vital components of clinical excellence in nephrology.

Recent research suggests that patients with chronic kidney disease have a limited understanding about treatment options, [6] and they report infrequent conversations about end of life care with their nephrologists [7]. Similarly, a survey of nephrology fellows revealed that they receive little formal training in communication skills and end of life care [8, 9]. To this end, NephroTalk, a published framework and communication skills workshop has been shown to improve preparedness among nephrology fellows to deliver bad news, express empathy, and discuss matters such as dialysis withdrawal [10].

### Professionalism and humanism

Patients with CKD rely on their nephrologists not just for medical treatment, but also for holistic care including “psychosocial and spiritual support” [7]. Caring, compassion, and empathy are elements of humanism that are not only necessary at the time of diagnosis of kidney ailments, but are also indispensable over time as patients endure the lifelong challenges associated with their chronic illness. Clinically excellent nephrologists are generous with time, deeply caring and dedicated. Issues related to professionalism emerge regularly in the nephrologist-patient relationship, especially along the lines of autonomy, social justice, and the primacy of patient welfare [11].

Interviews and focus groups with female renal patients found that many felt that their physicians could be more professional and humanistic. According to one participant, “We don’t need other numbers. Creatinine, Kt/V, and hemoglobin here are enough.” Another argued, “The life of a person with a chronic disease is entangled with the disease and with the fear of the disease. It’s impossible to separate our life, our lovers, our failures, our families, our dreams and our diseases. When I’m mad at the world, my creatinine goes up…But how many doctors do you know, who you could talk about this with?” [12]. This work reminds us that nephrologists need to go beyond merely providing medical treatment to their patients.

Mr. K was a bedbound 65-year old patient with ESRD on HD who had multiple comorbidities and was rapidly declining [13]. He had come to the conclusion that he was “ready to die” and sought a hospice. Hospice turned him down because he wanted to continue dialysis, which he believed positively impacted his quality of life. Mr. K’s nephrologist lobbied to find him hospice care that would allow him continued HD, thereby respecting the patient’s preferences and goals of care.

The Arnold P. Gold Foundation (APGF) is dedicated to advancing humanism in medicine [14]. The Foundation’s highest honor is the annual ‘Leonard Tow Humanism in Medicine Award’ which is given to those who best demonstrate the Foundation’s ideals of outstanding compassion in the delivery of care; respect for patients, their families, and healthcare colleagues; and clinical excellence. Nephrologists have been the recipients of this award multiple times in recent years (personal communication with APGF, data not published).

### Knowledge

Given the constantly evolving nature of nephrology, it is paramount for nephrologists to stay current with research advancements and with the new guidelines/care protocols. Withdrawal of treatment with HD for those whom the side effects may outweigh the benefit is an example of an area where evidence has advanced significantly in recent years [15, 16]. Given that withdrawal of dialysis for many patients is a core component of ethical and dignified clinical care [17], the Renal Physician Association (RPA) and American Society of Nephrologists (ASN) have issued joint clinical practice guidelines (first in 2000 [18] and an updated edition in 2010 [19]) to assist nephrologists in decision-making on the initiation and termination of dialysis. However, merely 61 % of nephrologists belonging to RPA were even aware of the RPA/ASN guidelines, and only 51 % of these doctors reported using them [20].

An Australian study of patients, trainees, and nephrologists set out to ascertain the attributes of the superior nephrologist [21]. Not surprisingly, a strong knowledge base was unanimously identified as a critical element. A recent analysis of academic roles of nephrologists in graduate medical education identified that many have core program leadership roles in medical residency programs, participating as advisors, lecturers, research mentors, and inpatient attending [22].

### Skillful negotiation of the health care system

The steady rise of healthcare costs and the impending physician shortage have been well documented and seriously threaten the viability of the American healthcare system. The field of nephrology has been especially burdened. Overall Medicare expenditures on ESRD increased from $12.3B in 2000 to $28.6B in 2012 [23]. Meanwhile, the number of patients with CKD continues to increase [24], even as medical school students’ interest in nephrology is declining [25]. Given this incongruity, new healthcare delivery models are hoping to alleviate some of the challenges. Effective use of appropriate resources distinguish good from excellent nephrologists.

Interdisciplinary models which include an infrastructure of dedicated renal pharmacists, care gap nurses and nephrology specialty specific case managers that allow the health care system to identify patients at risk and help close gaps in their health care are hoping to enhance the quality of care while mitigating costs in nephrology. The key stake holders in this interdisciplinary care model-patients, nephrologists, dialysis providers, health insurers and federal government recognize that changes are needed to deliver quality care and improve safety.

Whereas the canonical model of care for a condition like CKD involves a 2-person PCP-nephrologist partnership, the interdisciplinary approach spreads the responsibilities of care among several specialists (e.g., an advanced practitioner, kidney specialist, pharmacist, etc.) [26]. As the interdisciplinary model heavily increasingly emphasizes the importance of a coordinated team, the precise boundary lines between practitioners’ scope of care can become blurred. These coordinated teams attempt to serve patient-centered care and minimize the chance of medical errors. Interdisciplinary models strive to increase patient engagement in their own care. By incorporating additional providers such as social workers and dieticians into the treatment team, patients are educated and empowered to make lifestyle changes that will ultimately improve their health and prognosis. For instance, a 2007 Canadian study found that the 187 CKD patients who underwent educational sessions and worked with a clinic nurse, social worker, and dietician had improved survival outcomes compared to similar patients who experienced traditional care [27]. A larger 3-year prospective study conducted in Taiwan, comparing traditionally managed patients with CKD versus those cared for interdisciplinary teams (a nephrologist leader along with nurse educators, dieticians, social workers, and pharmacy specialist, and surgeon), found a 51 % reduction in patient mortality among those receiving team-based care [28]. Having nephrologist who are skilled in the negotiation of our complex healthcare system serve as the glue holding the team together and the quarterback carrying out the complex game plan may augment the chances of a successful outcome [29].

### Diagnostic acumen

Nephrologists who excel in diagnostic reasoning are often summoned to tackle the toughest cases. Thoughtful reflection about patient stories and superior judgment are sine qua nons among nephrologists who are gifted diagnosticians. They have the ability to uncover or comprehend previously unforeseen features of the illness script. In the end, these masters are often able to draw sound conclusions and make sense of what seemed like noise to others.

A 45 year old Caucasian male initially presented to his primary care physician with fatigue and anorexia after a vacation to South Africa and Thailand [30]. His physical exam was unremarkable, and his blood counts including renal function were normal. After 6 months of progressive symptoms, including anorexia, fatigue, and an unintentional 10 kg weight loss, he returned to the hospital. In the 2 weeks preceding the admission, the patient experienced left forearm pain with numbness on the medial aspect of his left hand. He was found to have hepatitis B surface antigenemia. Over the following 2 weeks, he developed neurologic dysfunction in his right arm, then his right leg, and finally his left lower extremity. All the while, his systemic symptoms worsened and he developed new onset hypertension that was difficult to control, requiring four anti-hypertensive agents. His physical exam was most notable for muscle wasting, bilateral median nerve palsies, a left ulnar nerve palsy, and distal muscle weakness. His labs revealed an elevated white blood count, normal renal function, hypoalbuminemia, and elevated transaminases. Urinalysis showed microscopic hematuria and his 24 h urine had 4.7 gram of protein. Serologies revealed a hepatitis B viral DNA load of 5.03 log IU/ml, and negative cryoglobulins, hepatitis C, syphilis, anti-nuclear and anti-cytoplasmic antibodies. His serum complements, serum and urine protein electrophoresis were normal. One day during the course of the hospitalization, he developed sudden abdominal pain and underwent a CT angiogram of his mesenteric and renal vasculature which revealed bilateral segmental renal infarctions. When nerve conduction studies confirmed the acute motor and sensory axonopathies, a sural nerve biopsy was performed that had findings consistent with a medium vessel vasculitis. A diagnosis of polyarteritis nodosa was made based on the clinical presentation, the serologic evidence of acute hepatitis B seroconversion, and the medium vessel vasculitis seen on nerve biopsy. He was treated with glucocorticoids, lamivudine, and tenofovir. Six months after diagnosis, he had resolution of his proteinuria, and undetectable hepatitis B viral DNA.

In this case, the clinicians were not only thorough but they modeled superlative diagnostic acumen by relating the renal infarctions, nerve biopsy findings and acute hepatitis B infection to make a unifying diagnosis.

### Scholarly approach to clinical practice

With continual advances in clinical and basic science research, clinically excellent nephrologists must take a scholarly approach to clinical practice so as to be responsive to new concepts, protocols, and paradigms. Beyond keeping up to date, one must be skilled applying this information judiciously when making clinical decisions. Many also choose to contribute to the generation of new knowledge in the field. The initiation of a screening protocol for polyoma BK virus (PBKV), which sought to decrease rates of post-renal-transplantation viral disease, can exemplify being scholarly in approaching clinical practice.

Although PBKV infects approximately 90 % of Americans by adulthood, it is usually asymptomatic and can remain latent in urologic epithelium. In the setting of renal transplantation, the immunosuppressive agents used to prevent allograft rejection allow for reactivation of PBKV with shedding of virus into urine. Left unchecked, viruria can lead to viremia and nephropathy with subsequent graft loss in 10–50 % of cases [31]. Allografts can be salvaged with timely reduction of immunosuppression. In 2005, an international panel of experts (including nephrologists) published evidence based recommendations for screening, diagnosis, and management of PBKV nephropathy [32].

Given these recommendations and increasing urgency to develop some type of way to mitigate or prevent PVN, there was a growing consensus that regular screening should be implemented. Clinicians at Albany Medical College established best practices for promptly reducing immunotherapy intensity in the 16 % of their patients screening positive [33]. Not only did none of their patients progressed to develop PBKV nephropathy, but also their patients averted other viral infections like CMV or EBV too. Further, by noting that PBK tends to reactivate in immunosuppressed patients, Albany clinicians actively sought to generate new insights by investigating whether polyoma viremia could be employed as surrogate marker for overimmunosupppression. In sum, these clinicians were not only applying the most up-to-date knowledge but they also actively moved the field forward.

### Exhibiting passion for clinical medicine

Nephrology is a multifaceted specialty offering home to eclectic interests including experts in dialysis, transplantation medicine, hypertension, and immunology – to name a few. Among clinically active nephrologists, caring for patients with chronic kidney disease and dialysis accounts for over 90 % of their effort [21]. As such, a vast majority of patients referred to nephrologists are likely to remain connected with their clinician for prolonged time periods. To illustrate passion, which can be defined as a deep enthusiasm, the biography of Thomas Addis (1881-1949) written by the Nobel Laureate Linus Pauling, as well as Addis’ own book on Glomerular Nephritis can serve as exemplary testimonials [34–36]. Thomas Addis is renowned for his many contributions to nephrology ranging from counts of urinary cells to the impact of diet in chronic kidney disease. Addis ingeniously followed his patients meticulously throughout the course of their lifelong disease and he emphasized the importance of continuity of care and the therapeutic alliance between patients and physicians. Addis is known for tailoring prescriptions and frequency of clinic visits to his patients at each phase of the disease. Ahead of his time, Addis also stressed the importance of knowing the patient as a person or their personal histories. Arthur Bloomfield noted that his relations with his patients were marked by “deep friendship and concern.” Although a consummate researcher, according to Ray Wilbur, Addis was, committed “not [to] research just for research’s sake, but to [relieving] human suffering.” Before dialysis or transplantation when all patients died of renal disease, (32) Addis found it very difficult to visit his patients when the end was near. They would, however, not be satisfied with any of his associates, so in the end he would see them and provide what comfort he could. “It is our job to do our best to keep [the patients] on the firing line to the very last gasp. Since our best endeavor amounts to almost nothing, we need not take ourselves too seriously. . . . We retreat to the wings to watch the last act of the tragedy.”

Some of the greatest passion in contemporary medicine can be witnessed by those who share their enthusiasm daily through social platform. Medical blogging has become increasingly common, and many nephrologists are prominent in the renal blogosphere [37–39].

## Summary

Many examples of clinical excellence in renal medicine can be found in the published medical literature. Nephrology largely focuses on improving the quality of life of patients with diseases brought on by kidney dysfunction. Nephrologists often encounter patients who require care for the duration of their lives and thus long term doctor-patient relationships develop. Optimal nephrology care requires clinical skills across the domains of clinical excellent delineated in this manuscript. These domains of clinical excellence, described by the Miller-Coulson Academy of Clinical Excellence, apply very well to the field of nephrology. We were able to find multiple examples for each domain, and there were not any cases that we reviewed which unearthed a feature of clinical excellence that could not be captured our holistic definition.

In this expanding series of papers that highlights cases of exemplary care from the literature; it may be worth stating that a different team of researchers writing a similar manuscript may have chosen examples distinct from those presented in this article. This is not a methodological weakness but instead is a known expectation in more subjective and qualitative works.

The examples presented in this article may trigger a physician’s memory to recall situations in which she was clinically excellent, thereby bolstering her confidence and positive self-image. This introspection can serve to remind us all of the success that can result when one is committed to excellence and the *adjacent possible* [40].

## Abbreviations

MCACE: Miller Coulson Academy of Clinical Excellence
HD: Hemodialysis
CKD: Chronic kidney disease
ESRD: End stage renal disease
APGF: Arnold P Gold Foundation
RPA: Renal physicians association
ASN: American Society of Nephrology
PBKV: Polyoma BK virus
